# Regulation of Human Adenovirus Replication by RNA Interference

**Published:** 2015

**Authors:** N. A. Nikitenko, T. Speiseder, E. Lam, P. M. Rubtsov, Kh. D. Tonaeva, S. A. Borzenok, T. Dobner, V. S. Prassolov

**Affiliations:** Engelhardt Institute of Molecular Biology, Russian Academy of Sciences, Vavilova Str., 32, Moscow, 119991, Russia; Heinrich Pette Institute – Leibniz Institute for Experimental Virology, Martinistrasse 52 D-20251, Hamburg, Germany; S.N. Fedorov Eye Microsurgery Complex of the Ministry of Health of the Russian Federation, Beskudnikovskiy Blvd., 59A, Moscow, 127486, Russia

**Keywords:** RNA interference, human adenoviruses, small interfering RNAs, lentiviral vectors, small hairpin RNAs

## Abstract

Adenoviruses cause a wide variety of human infectious diseases. Adenoviral conjunctivitis and epidemic keratoconjunctivitis are commonly associated with human species D adenoviruses. Currently, there is no sufficient or appropriate treatment to counteract these adenovirus infections. Thus, there is an urgent need for new etiology-directed therapies with selective activity against human adenoviruses. To address this problem, the adenoviral early genes *E1A *and *E2B *(viral DNA polymerase) seem to be promising targets. Here, we propose an effective approach to downregulate the replication of human species D adenoviruses by means of RNA interference. We generated *E1A *expressing model cell lines enabling fast evaluation of the RNA interference potential. Small interfering RNAs complementary to the *E1A *mRNA sequences of human species D adenoviruses mediate significant suppression of the *E1A *expression in model cells. Furthermore, we observed a strong downregulation of replication of human adenoviruses type D8 and D37 by small hairpin RNAs complementary to the *E1A *or *E2B* mRNA sequences in primary human limbal cells. We believe that our results will contribute to the development of efficient anti-adenoviral therapy.

## INTRODUCTION

Adenoviral ocular infections are of great current concern in biomedicine due to the wide prevalence and high rate of adenovirus infection episodes. Adenoviruses commonly cause human respiratory and gastrointestinal infections, adenoviral conjunctivitis (inflammation of the conjunctiva) and epidemic keratoconjunctivitis (combined inflammation of the cornea and conjunctiva) [1]. In Russia, more than 300,000 people are annually diagnosed with epidemic keratoconjunctivitis [2]. The most serious eye infections are caused by human species D adenoviruses (HAdVs) types D8, D19, and D37 [3]. HAdV infection affects people of all age groups [4]. In Russia, about 18 million people visit the ophthalmologist for inflammatory eye diseases annually, which accounts for up to 80% of temporary disability due to eye diseases and 10–30% of visual acuity loss and blindness [5]. An epidemic spread, large loss due to temporary disability, a high likelihood of complications (temporary or permanent loss of vision), and a multitude of clinical manifestations define the medical and social significance of adenoviral eye diseases. Adenovirus infections present a serious health risk for immunocompromised individuals [6]. Obesity in children and nonalcoholic fatty liver disease in adults are known to be caused by human spicies D adenovirus type 36 (HAdV D36) [7].

Currently, the limited efficacy of therapies for the adenovirus infection [8] dictates the need for developing drugs with selective activity against adenovirus pathogens.

Human adenovirus early genes, such as the DNA polymerase *E2B *gene and *E1A *gene, which are involved in viral DNA replication, seem to be potential targets for antiviral therapy [9-11].

*E1A *gene products promote the G0 to S phase cell cycle transition. Interaction between *E1A *gene products and the retinoblastoma protein (pRb) activates the E2F1 transcription factor, triggering expression of the genes necessary for progression through the S phase. This enables the adenovirus to replicate in the infected cell using the host’s replication machinery. One of the functions of the E2F1 transcription factor is transactivation of the p14/ARF protein that is accompanied by induction of p53-dependent apoptosis. During viral infection, the 55 kDa E1B protein inactivates p53, thereby preventing induction of apoptosis in the cell until the adenovirus replication cycle is completed [12, 13].

Small interfering RNAs (siRNAs), which are short 21–23 bp duplexes with 2–3 overhanging nucleotides at 3’-ends capable of suppressing gene expression at the post-transcriptional level, are commonly used in gene function studies [14, 15]. Interfering RNAs were shown to be capable of suppressing the expression of various target genes, including viral genes [16-18]. Interfering RNAs could be effectively delivered to cells by recombinant lentiviral vectors encoding small hairpin RNAs (shRNAs), which are precursors of small interfering RNAs [19-21].

We believe that this approach can be used for downregulation of the *E1A and E2B *expression [10, 11] of HAdVs causing ocular and respiratory tract infections.

This paper presents the results of *E1A *gene expression downregulation in human species D adenoviruses types D8, D19, D36, and D37 using siRNAs and shRNAs, as well as inhibition of HAdVs D8 and D37 replication upon simultaneous downregulation of *E1A *and* E2B *expression using shRNAs.

## MATERIALS AND METHODS

**Cell culture**

Human embryonic kidney cells HEK293 [22], human lung adenocarcinoma epithelial cells A549 (DSMZ ACC107; Brauschweig, Germany), human nonsmall cell lung cancer cells H1299, A549 E1A and H1299 E1A model cells were grown in a DMEM medium (Life Technologies, UK) containing 10% fetal bovine serum (Life Technologies), 4 mM*L*-glutamine, 1 mM sodium pyruvate, and streptomycin/penicillin at a concentration of 100 μg/mL and 100 U/mL, respectively, at 37°C under 5% CO_2_ atmosphere. Primary human limbal cells [24, 25] were cultured on a DMEM/F12 medium (Life Technologies) containing 10% fetal bovine serum (Life Technologies), 4 mM*L*-glutamine, 1 mM sodium pyruvate, 10 mM HEPES, 0.4 μM insulin, 10 nM dexamethasone, and streptomycin/penicillin at a concentration of 100 μg/ mL and 100 U/mL, respectively, at 37°C under 5% CO_2_.

**Lentiviral vectors**

Recombinant lentiviral vectors were constructed using standard genetic engineering techniques [26, 27]. Recombinant lentiviral virions were generated in the HEK293 cells by calcium phosphate co-transfection of lentiviral vector DNA and plasmids directing the synthesis of all the lentiviral proteins required to produce infectious lentiviral particles. Infectious pseudoviral particles were collected during 2 days with 12-h intervals. A549 and H1299 cells were used for lentivirus titer estimation. Viral stocks with titers of 5 × 10^5^ to 5 × 10^6^ were further used.

**Model cell lines**

Model cells expressing the *E1A *gene of the human adenovirus type 36 (*E1A-D36*) were produced by transduction of A549 and H1299 cells with LeGO-iGT-Puroopt- based pseudolentiviral particles E1A-LeGO-iGT containing an expression cassette “promoter–*E1A *gene of HAdV D36–IRES–*dTomato *marker gene –puromycin resistance gene.”

In order to generate a H1299 shE1A model cell line, the initial H1299 cells were transduced with lentiviral particles containing a sequence encoding shE1A, the* Cerulean *fluorescent protein gene, and the blasticidin (BSD) resistance gene. 48 h post transduction, the cells were placed into a selective medium with 5 μg/mL blasticidin. Selection was conducted for 10 days. H1299 shE1A cells were then analyzed by flow cytometry.

Limb shE2B, Limb shE1A, and LimbshE2B/shE1A model cell lines were prepared by transduction of primary human limbal cells with pseudolentiviral particles encoding shE2B [10, 11] or shE1A.

**siRNAs**

We designed siRNAs complementary to different regions of the *E1A *mRNA sequences of HAdVs types 8, 19, 36, and 37. xpression. To suppress the target gene expression the following 21 bp siRNAs were synthesized (Sintol, Russia): siE1A-1 (sense strand 5’-GGAGGACUUUGUGAAUACAUU- 3’, antisense strand 5’-UGUAUUCACAAAGUCCUCCUU- 3’);siE1A-2(sense strand 5’-GAGGCUGUGAAUUUAAUAUUU-3’, antisense strand 5’-AUAUUAAAUUCACAGCCUCUU-3’), and siE1A-3 (sense strand 5’-GCUCUGUGUUACAUGAAAUUU- 3’, antisense strand 5’-AUUUCAUGUAACACAGAGCUU- 3’). siScr having no homology with known viral mRNAs as well as human, mouse, and rat mRNAs (sense strand 5’-CAAGUCUCGUAUGUAGUGGUU-3’, antisense strand 5’-CCACUACAUACGAGACUUGUU- 3’) were used as a control. The siRNAs were designed using the Whitehead Institute siRNA Selection Program [28].

**siRNA transfection**

Cells in the exponential growth phase were seeded into 24-well plates, 3 × 10^4^ cells per well, 1 day prior to the experiment and transfected with siRNAs at a concentration of 200 nM using a Lipofectamine® 2000 Transfection Reagent (Life Technologies) according to the manufacturer’s protocol.

**shRNAs**

We constructed shE1A-LeGO-Cer/BSD and shScr- LeGO-Cer/BSD lentiviral vectors encoding shRNAs: shE1A, which corresponds to siE1A-1 (sense strand 5’-p-aacgATATTAAATTCACAGCCTCcttcctgcaaGAGGCTGTGAATTTAATATtttttc- 3’, antisense strand 5’-p-tcgagaaaaaATATTAAATTCACAGCCTCttgcaggaagGAGGCTGTGAATTTAATATcgtt- 3’) and a control shScr (sense strand 5’-p-gatccgCCACTACATACGAGACTTcttcctgtcaCAAGTCTCGTATGTAGTGGtttttg- 3’, antisense strand 5’-p-aattcaaaaaCCACTACATACGAGACTTGtgacaggaagCAAGTCTCGTATGTAGTGGcg- 3’). In this work, we also used lentiviral vectors shE2B-LeGO-G (encoding shRNA targeting the DNA polymerase mRNA of HAdVs D8, D19, D36, and D37) and shScr-LeGO-G, which were described previously in [10, 11].

**Flow cytometry**

The cell fluorescence intensity was measured by an Epics 4XL flow cytometer (Beckman Coulter, USA). Obtained results were analyzed with WinMDI software, version 2.8

**Real-time PCR**

Total RNA was isolated from cell cultures using a TRIzol® reagent (Life Technologies) according to the manufacturer’s protocol. Reverse transcription was carried out using ImProm-II™ Reverse Transcriptase (Promega, USA). *E1A*-*D36 *mRNA levels were assessed in real-time PCR using specific primers : sense sequence 5’-GCATCCAGAGCCATTTGAGC-3’; antisense sequence 5’-TTAGGGTCGTCATCATGGGC-3’. Resulting values for each sample were normalized to the β-actin housekeeping gene expression. The β-actin expression level was quantified using the following primers: sense sequence 5’-ATGGATGATGATATCGCCGC- 3’and antisense sequence 5’-CTTCTGACCCATGCCCAC- 3’. Real-time PCR was performed in 96-well plates using a MiniOpticon system (Bio-Rad, USA) and an iQ SYBR Green Supermix reagent (Bio- Rad) in accordance with the manufacturer’s recommendations. PCR products were analyzed using the MiniOpticon system software (Bio-Rad).

**Quantification of the human adenovirus genome copy number**

HAdV D8 (ATCC® VR-1085AS/RB™ ATCC® VR- 1085AS/RB™) and HAdV D37 (ATCC® VR-929™) were purchased from the American-Type-Culture-Collection (ATCC). Replication of species D human adenoviruses was evaluated 6 days post infection. For this, HAdV D8 and D37 infected cells were harvested, and total DNA was isolated using a QIAamp DNA Mini Kit (QIAGEN, Germany) according to the manufacturer’s recommendations. Quantitative PCR was performed as described in [29] on a Rotor Gene Q cycler (QIAGEN) using a TaqMan® Universal PCR Master Mix reagent (Life Technologies). PCR products were analyzed using the Rotor Gene Q cycler software (QIAGEN).

**Statistical data processing**

All data are presented as a mean ± standard deviation (SD). The statistical significance was determined using the unpaired two sample t-test and GraphPad Prism software 6 (GraphPad Software, USA). The value of* p* < 0.05 was considered statistically significant.

## RESULTS AND DISCUSSION

**Derivation of model cell lines expressing the HAdV D36 *E1A *gene**

Model cell lines A549 E1A and H1299 E1A expressing the E1A gene of human species D adenovirus type 36 (*E1A-D36*) were obtained in order to analyze the functional activity of synthetic siRNAs and lentiviral vectors directing the synthesis of shRNAs in transduced cells.

**Fig. 1:**
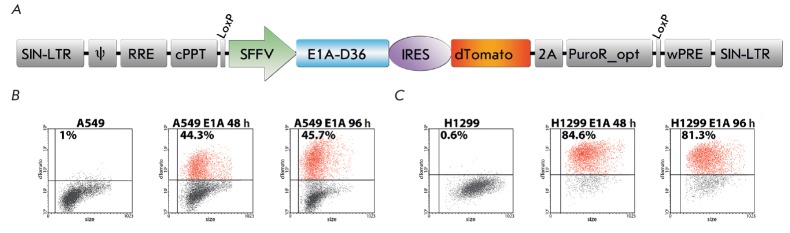
Model cell lines. A – Lentiviral vector containing the *E1A-D36 *gene of HAdV D36 and the dTomato/puromycin- resistance fusion gene separated by an IRES sequence. The vector is based on the LeGO-iGT-Puro-opt vector. SIN-LTR– self-inactivating-long-terminal repeat ; ψ – packaging signal; RRE– rev-responsive element; cPPT– central polypurine tract; SFFV– spleen focus-forming virus U3 promoter; *E1A-D36*– *E1A *gene of HAdV D36; IRES– internal ribosome entry site; dTomato – dTomato fluorescent protein; 2A– self-cleaving peptide of the porcine teschovirus-1; PuroR_opt– codon optimized cDNA of puromycin resistance; wPRE– Woodchuck hepatitis virus post-transcriptional regulatory element. B – Flow cytometry assay of A549 E1A cells expressing the dTomato fluorescent protein. C – The number of H1299 E1A cells expressing the dTomato fluorescent protein measured by flow cytometry

The *E1A *expression in cells is known to induce p53- dependent apoptosis [30]. *E1A *expression products promote the G_0_ to S phase transition and disturbance of the mechanisms controlling DNA synthesis. This increases the likelihood of DNA damage during replication. In response to heavy DNA damage, the p53 protein triggers a cascade of reactions leading to apoptosis. Therefore, A549 and H1299 cells were first transfected with siRNAs complementary to various fragments of the *E1A *mRNA and with control siScr. After 24 h, the cells were transduced by pseudolentiviral particles (*Fig. 1A*) containing the expression cassette “promoter– target *E1A-D36 *gene–IRES–dTomato fluorescent protein gene–puromycin resistance gene.” The marker gene encoding the dTomato fluorescent protein and the target *E1A *gene are separated by the IRES sequence (internal ribosome entry site). IRES allows for the synthesis of several proteins from a single mRNA in eukaryotic cells. Thus, the target and marker genes are expressed with a comparable efficiency [31]. This enables indirect estimation of the *E1A *expression level via measuring dTomato fluorescence by flow cytometry.

The efficiency of the introduced genes, expression was evaluated by flow cytometry detection of dTomato fluorescence and real-time PCR. The flow cytometry data for evaluation of the transduction and subsequent transgene expression efficiency are shown in *Fig. 1B* and *Fig. 1C*.

The number of cells of the A549 E1A and H1299 E1A model lines in which dTomato protein fluorescence was registered amounted to 44 and 85% of the total cell population, respectively, compared to a control (nontransduced A549 and H1299 line cells).

These findings indicate that the produced A549 E1A and H1299 E1A transgenic model cells express the introduced target gene and *dTomato *marker gene with a high efficiency.

According to the obtained results, the number of fluorescent cells in A549 E1A and H1299 E1A model lines is significantly different. The heterogeneity of A549 E1A and H1299 E1A cells in the *dTomato *expression level may be caused by individual properties of the cells. The different number of lentiviral provirus molecules is integrated in the genome of transgenic cells. The viral DNA integrates into different regions of the genome, which may provide varying transgene expression levels. Despite the similarity of the morphology and origin of A549 and H1299 cells, differences between these two lines may be one of the causes. The* E1A *gene expression is known to result in p53-dependent apoptosis. H1299 cells are p53-deficient. Hence, the likelihood of apoptosis in H1299 E1A cells is significantly lower than in A549 E1A cells. It may be assumed that A549 cells are transduced with the same efficiency as H1299 cells, but p53-dependent apoptosis occurs in most of the transduced A549 E1A cells, enabling detection of dTomato fluorescence in a smaller number of A549 E1A cells compared to H1299E1A model cells.

**siRNA-mediated downregulation of *E1A-D36 *gene expression**

The structure of the expression cassette “promoter–* E1A-D36 *gene–IRES–*dTomato *marker gene –puromycin resistance gene” enables rapid assessment of the silencing activity of siRNAs and the lentiviral vectors directing the synthesis of shRNAs (precursors of siRNAs).

Because of degradation of a mRNA common to both genes due to the inhibitory activity of interfering RNAs targeting *E1A-D36 *mRNA, the cell stops producing both the *E1A-D36 *gene products and the dTomato fluorescent protein, which can be quantified by flow cytometry.

Since the *E1A *gene expression results in induction of apoptosis, the experiment was performed as follows: siRNAs complementary to various regions of the* E1A-D36 *mRNA were transfected into A549 and H1299 cells; after 24 h, the cells were transduced with pseudolentiviral particles containing an expression cassette “promoter SFFV–*E1A-D36 *gene–IRES–dTomato fluorescent protein gene–puromycin resistance gene.” The efficiency of siRNAs was evaluated by flow cytometry and real-time PCR 48 and 96 h post transduction.

**Fig. 2:**
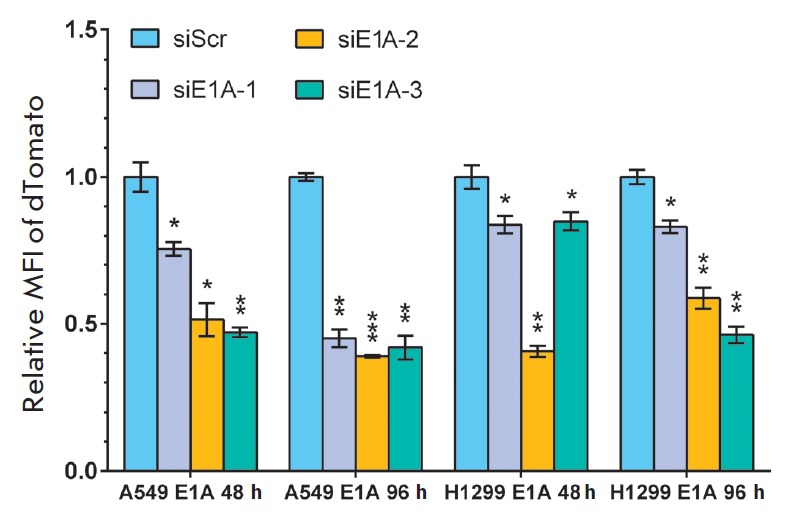
Silencing activity of siRNAs complementary to the *E1A-D36 *mRNA sequence. The silencing activity of siRNAs targeted against the *E1A-D36 *mRNA resulted in a reduction in the dTomato MFI in A549 E1A and H1299 E1A cells. 1 corresponds to the dTomato MFI value in cells transfected with siScr. All reported values are a mean ± standard deviation of three independent experiments. The differences between siScr and targeting siRNAs were statistically significant in all cases (^*^p < 0.05, ^**^p  < 0.01, ^***^p  < 0.001)

The activity of siRNAs complementary to the *E1AD36* mRNA caused a significant decrease in the *dTomato* expression level (*Fig. 2*). In the population of A549 E1A cells transfected with siE1A-1, siE1A-2, and siE1A-3, the mean fluorescence intensity (MFI) of dTomato was decreased by 25, 49, and 53% after 48 h and by 55, 61, and 58% after 96 h, respectively, compared to control A549 E1A cells transfected with siScr. A siRNA biological activity assay showed that MFI of the dTomato fluorescent protein in the H1299 E1A model cell population was decreased by 18, 60, and 17% under the action of siE1A-1, siE1A-2, and siE1A-3 as early as after 48 h and by 18, 44, and 56% after 96 h, respectively, compared to control H1299 E1A cells transfected with siScr. All the presented data were obtained in three independent experiments (*p* < 0.05).

**Fig. 3:**
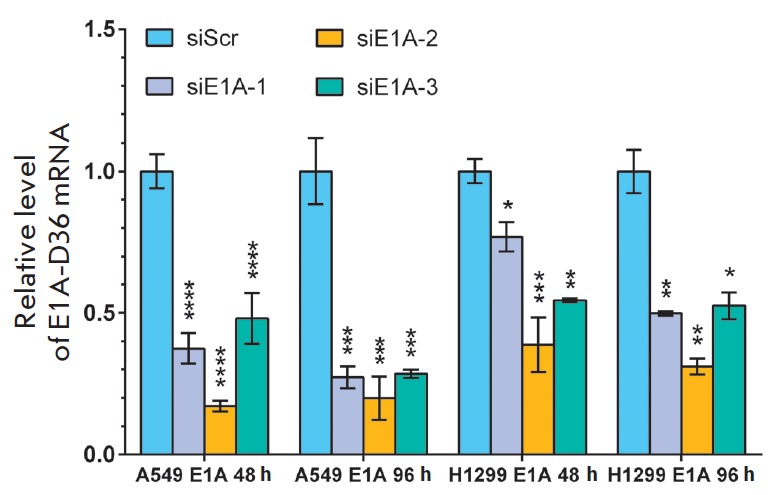
siRNA-induced downregulation of *E1A-D36* expression. Levels of *E1A-D36 *mRNA in A549 E1A and H1299 E1A model cells were analyzed by RT-qPCR. 1 corresponds to values for the cells transfected with siScr. Results were normalized to the endogenous b-actin mRNA level. All reported values are a mean ± standard deviation of three independent experiments. The differences between siScr and targeting siRNAs were statistically significant in all cases (^*^p  < 0.05, ^**^p  < 0.01, ^***^p < 0.001, ^****^p  < 0.0001)

Results obtained by flow cytometry are consistent with the real-time PCR data. The effect of siRNAs on the *E1A-D36 *mRNA expression level in model cells was assessed 48 and 96 h post transduction with pseudolentiviral particles encoding the target gene (*Fig. 3*). The *E1A-D36* mRNA expression level in A549 E1A cells transfected with siE1A-1, siE1A-2, and siE1A-3 was reduced by 58, 83, and 63% and 69, 88, and 72% 48 h and 96 h post transduction, respectively, compared to control cells. The *E1A-D36 *mRNA level in H1299 E1A model cells transfected with siE1A-1, siE1A-2, and siE1A-3 was reduced by 28, 71, and 46% and 50, 69, and 47% 48 h and 96 h post transduction, respectively, compared to control cells.

A reduction in the mean fluorescence intensity of the dTomato marker protein occurs more slowly and less effectively than suppression of target gene expression at the mRNA level. These data may be explained by the fact that the fluorescent protein is quite stable, and its half-life is about 72 h.

According to the flow cytometry and real-time PCR data, the *E1A-D36 *gene expression was mostly affected by siE1A-2. The siRNA silencing activity depends on several factors. These include the secondary structure of a target mRNA, siRNA sequence uniqueness, and stability and the thermodynamic asymmetry of siRNA duplexes. The secondary structure of a target mRNA or the proteins bound to it are known to be capable of hindering access for siRNAs [15, 32].

**Downregulation of *E1A-D36 *gene expression by a lentiviral vector encoding shRNA**

**Fig. 4:**
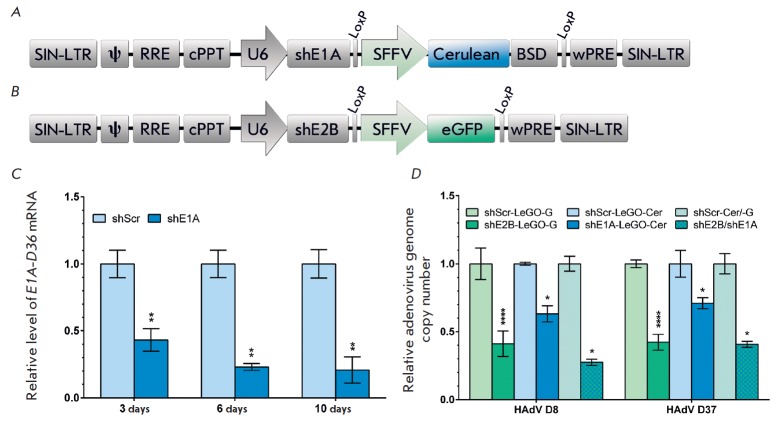
Silencing activity of shRNAs targeting *E1A *and *E2B *(DNA-polymerase) mRNAs of human species D adenoviruses. A – LeGO-Cerulean/BSD-based lentiviral vector encoding shE1A complementary to *E1A *of HAdVs D8, D19, D36, and D37. B – LeGO-G-based lentiviral vector encoding shE2B complementary to *E2B *of HAdVs D8, D19, D36, and D37. SIN-LTR– self-inactivating-long-terminal repeat; ψ– packaging signal; RRE– rev-responsive element; cPPT– central polypurine tract; U6– murine U6 pol-III promoter; SFFV– spleen focus-forming virus U3 promoter; Cerulean– fluorescent protein Cerulean; eGFP– enhanced green fluorescent protein; BSD– blasticidin resistance gene; wPRE– Woodchuck hepatitis virus post-transcriptional regulatory element. C – RT-PCR quantification of E1A-D36 mRNA levels in H1299 cells expressing shE1A 3, 6, and 10 days post transduction with a lentiviral vector encoding E1A-D36. Results were normalized to the endogenous b-actin mRNA level. 1 corresponds to values for the control cells expressing shScr. All reported values are a mean ± standard deviation of three independent experiments. The differences between shScr and shE1A were statistically significant in all cases (^**^p < 0.01). D – Downregulation of HAdV D8 and D37 genome replication in primary human limbal cells. Viral genome replication was measured via RT-qPCR. 1 corresponds to values for the control cells. All reported values are a mean ± standard deviation of three independent experiments. The differences between shScr and targeting shRNAs were statistically significant in all cases (^*^p  < 0.05, ^****^p  < 0.0001)

We constructed lentiviral vectors encoding shRNAs corresponding to siE1A-2 (with the highest suppression of *E1A-D36 *expression) and control siScr. Lentiviral vectors ensure integration of a sequence encoding shRNA into the cell genome, providing long-term suppression of the target gene expression. These lentiviral vectors were constructed based on the Le-GO-Cerulean/BSD (*Fig. 4A*) carrying the Cerulean fluorescent protein gene and blasticidin resistance gene. The shScr-LeGO-Cerulean/BSD vector encoding a hairpin structure having no homology with known viral mRNAs as well as rat, mouse, and human mRNAs was used as a control to demonstrate the absence of a nonspecific activity of shRNAs.

These lentiviral vectors were transduced into the genome of H1299 cells. Ten days after selection on blasticidin, the transduction efficiency of the H1299 shE1A and H1299 shScr cells was evaluated by flow cytometry of Cerulean reporter protein fluorescence. The number of fluorescent cells amounted to 92–98% of the total cell number in the population compared to control cells (non-transduced H1299 cells).

Then, H1299 shE1A model cells were transduced with E1A-LeGO-iGT pseudolentiviral particles. The biological activity of shE1A was evaluated 3, 6, and 10 days post transduction using real-time PCR in three independent experiments (*p* < 0.05). The expression level of the *E1A *target gene was down by 57, 77, and 80% under the action of shE1A after 3, 6, and 10 days, respectively, compared to the control (*Fig. 4C*).

We demonstrated that lentiviral vectors encoding shRNAs significantly suppress the *E1A-D36 *target gene expression. It should be noted that persistent suppression of the *E1A-D36 *expression mediated by shRNA-expressing lentiviral particles was preserved in model cells for 10 days. This demonstrates the high efficiency of the constructed lentiviral vectors.

**Downregulation of human species D adenoviruses replication by shRNAs in primary human limbal cells**

We evaluated the ability of shRNAs targeting mRNAs of the *E1A *and *E2B *HAdV early genes to suppress viral replication. Primary human limbal cells (Limb) were transduced with shE2B-LeGO-G (*Fig. 4B*) and shE1A-LeGO-Cerulean/BSD lentiviral vectors encoding shE2B and shE1A, respectively. Limbal cells transduced with pseudolentiviral particles carrying shScr (whose sequence has no homology with known viral mRNAs and mouse, rat, and human mRNAs) were used as a control.

Cells of the derived lines were infected with HAdV D8 and HAdV D37, which cause epidemic keratoconjunctivitis, at a multiplicity of infection of 20 fluorescence- forming units (FFU)/cell. The cells were cultured for 6 days post infection, which is sufficient to complete the full replication cycle of a human adenovirus. Six days post infection, the efficiency of shRNAs was assessed via qPCR to detect HAdV D8 and HAdV D37 genomes. We observed a significant downregulation of human adenovirus replication in primary human limbal cells. The copy number of HAdV D8 and HAdV D37 genomes was reduced by 59 and 58% under the action of shE2V, 37 and 30% under the action of shE1A, and 73 and 60% under the simultaneous action of shE2B and shE1A, respectively, compared to control cells (*Fig. 4D*).

We suppose that downregulation of expression of human species D adenoviral early genes results in premature termination of the adenovirus replication cycle. This explains the significant reduction in the adenovirus genome copy number. Nevertheless, we have not achieved a complete suppression of viral replication. This may be associated with the high multiplicity of infection (20 FFU/cell), the sample analysis time (6 days post infection, while maximum suppression of target gene expression by shRNAs is observed on the 9th–10th day (*Fig. 4C*) [10, 11]), as well as the low transduction efficiency of primary human limbal cells (50–70% fluorescent cells in the population according to fluorescence microscopy).

In the experiment, we used primary human limbal cells derived from the human cornea, which is affected in epidemic keratoconjunctivitis. We also used HAdVs D8 and D37 that are the main causative agents of the disease. Thus, we developed an *in vitro *model system of adenovirus ocular infection and demonstrated the high efficiency of shRNAs targeted against early genes of human spicies D adenoviruses.

## CONCLUSIONS

We developed an approach to efficiently suppress replication of human species D adenoviruses via RNA interference. In order to evaluate the silencing activity of siRNAs specific to the *E1A *gene of HAdVs D8, D19, D36, and D37, as well as lentiviral vectors that direct the synthesis of an analogous shRNA in cells, we produced model cell lines whose genome contains the expression cassette “promoter–*E1A-D36 *gene–IRES–* dTomato *marker gene– puromycin resistance gene.” These model cell lines enable rapid evaluation of the efficiency of interfering RNAs complementary to different regions of the target gene mRNA.

The high efficiency of these vectors in downregulation of human species D adenoviruses D8 and D37 replication was shown in primary human limbal cells. The simultaneous action of shE1A and shE2B led to a decrease in the adenovirus genome copy number by 70%, on average.

We believe that our findings will be helpful for the design and development of novel medicinal agents against human diseases caused by adenoviruses.

## Abbreviations

siRNA: small interfering RNA
shRNA: small hairpin RNA
HAdV: human adenovirus
FFU: focus forming unit
MFI: mean fluorescence intensity
